# Dynamic Change in Starch Biosynthetic Enzymes Complexes during Grain-Filling Stages in BEIIb Active and Deficient Rice

**DOI:** 10.3390/ijms231810714

**Published:** 2022-09-14

**Authors:** Yining Ying, Feifei Xu, Zhongwei Zhang, Piengtawan Tappiban, Jinsong Bao

**Affiliations:** 1 Institute of Nuclear Agriculture Science, College of Agriculture and Biotechnology, Zhejiang University, Zijingang Campus, Hangzhou 310058, China; ying_erin@163.com (Y.Y.); xuxufei@zju.edu.cn (F.X.); 12116001@zju.edu.cn (Z.Z.); piengtawan.tap@hotmail.com (P.T.); 2 Hainan Institute of Zhejiang University, Hainan Yazhou Bay Seed Lab, Yazhou Bay Science and Technology City, Yazhou District, Sanya 572025, China

**Keywords:** rice, starch synthase, starch branching enzyme, protein–protein interaction, protein complex, grain filling

## Abstract

Starch is the predominant reserve in rice (*Oryza sativa* L.) endosperm, which is synthesized by the coordinated efforts of a series of starch biosynthetic-related enzymes in the form of a multiple enzyme complex. Whether the enzyme complex changes during seed development is not fully understood. Here, we investigated the dynamic change in multi-protein complexes in an *indica* rice variety IR36 (wild type, WT) and its BEIIb-deficient mutant (*be2b*) at different developmental stages. Gel permeation chromatography (GPC) and Western blotting analysis of soluble protein fractions revealed most of the enzymes except for SSIVb were eluted in smaller molecular weight fractions at the early developing stage and were transferred to higher molecular weight fractions at the later stage in both WT and *be2b*. Accordingly, protein interactions were enhanced during seed development as demonstrated by co-immunoprecipitation analysis, suggesting that the enzymes were recruited to form larger protein complexes during starch biosynthesis. The converse elution pattern from GPC of SSIVb may be attributed to its vital role in the initiation step of starch synthesis. The number of protein complexes was markedly decreased in *be2b* at all development stages. Although SSIVb could partially compensate for the role of BEIIb in protein complex formation, it was hard to form a larger protein complex containing over five proteins in *be2b*. In addition, other proteins such as PPDKA and PPDKB were possibly present in the multi-enzyme complexes by proteomic analyses of high molecular weight fractions separated from GPC. Two putative protein kinases were found to be potentially associated with starch biosynthetic enzymes. Collectively, our findings unraveled a dynamic change in the protein complex during seed development, and potential roles of BEIIb in starch biosynthesis via various protein complex formations, which enables a deeper understanding of the complex mechanism of starch biosynthesis in rice.

## 1. Introduction

Rice (*Oryza sativa* L.) is one of the most important food crops worldwide. Starch, composed of amylose and amylopectin, is the most abundant component in the rice grain that provides nutrients to the developing embryo and seedling. The development of the rice endosperm starts after double fertilization, then the fertilized polar nuclei undergo mitotic cell proliferation and cellularization until 6 to 7 days after flowering (DAF), and it is at 5–20 DAF that genes encoding the enzymes of starch biosynthesis are vigorously expressed and accumulation of starch and other storage compounds occurs [1,2,3].

The starch biosynthesis pathway is orchestrated by multiple enzymes in rice endosperm, which mainly include ADP-glucose pyrophosphorylase (AGPase), granule-bound starch synthase (GBSS), soluble starch synthase (SSs), starch branching enzyme (BEs), starch debranching enzyme (DBEs), and phosphorylases (Phos). Seven AGPase subunits [4,5,6,7], eleven isoforms of SSs [8,9], three isoforms of BEs [10,11,12], four isoforms of DBEs [13,14,15], and two isoforms of Phos [16,17] were identified, and their functions in the process of starch synthesis have been elucidated by mutant and transgenic analyses. In addition, starch biosynthesis isozymes display as a complex form, rather than as monomers, to accomplish their activities in the starch biosynthesis pathway by associating with other enzymes. For example, starch plastidial phosphorylase (Pho1, also known as PHS1 and SP) form a complex with disproportionating enzyme (Dpe1) to use a broader range of sugars to synthesize malto-oligosaccharides (MOSs) and enhance the synthesis of long MOSs, which plays an important role in the initiation of starch synthesis [18]. Moreover, Pho1 may facilitate interactions between SSIIa and SSIIIa in amyloplasts; although, no direct association between SSIIa and SSIIIa was detected [19]. 

The direct evidence of physical interactions among starch biosynthetic enzymes was initially demonstrated in the developing endosperm of wheat [20]. Subsequently, further evidence for the identification of more phosphorylation-dependent complexes was accumulated in wheat [21], maize [22,23,24,25,26,27], barley [28], and rice [29,30,31,32]. Specifically, an approximately 230 kDa trimeric complex formed between SSI, SSIIa, and BEIIb is one of the best studied and well-characterized protein complexes, which is commonly found in cereals and contributes to the synthesis of short and intermediate amylopectin chains within the clusters [33]. A large protein complex (approximately 670 kDa) involving SSIII interacting with SSIIa, BEIIa, BEIIb, and several other proteins including pyruvate orthophosphate dikinase (PPDK) and AGPase has also been demonstrated in maize endosperm [23]. In addition, gel filtration analyses of soluble proteins from developing rice endosperm in a *japonica* background showed that rice starch biosynthetic enzymes, including SSI, SSIIa, SSIIIa, SSIVb, BEI, BEIIb, and PUL, form a larger protein complex (>700 kDa) than those found in other cereals [29].

BEs are essential for amylopectin biosynthesis because they are the only enzymes that introduce α-1,6 glycosidic bonds into α-polyglucans [34]. The three isoforms in rice (BEI, BEIIa, and BEIIb) play distinct roles, i.e., BEI transfers a variety of both short chains and intermediate chains (DP ≤ 40) while BEIIa and BEIIb preferentially form amylopectin short chains of DP 6-15 and DP 6-7, respectively [10,34]. Although BEI accounts for the largest relative activities (approximately 80%) [35], BEIIb (the major form of BEII in maize and rice) made the greatest contribution to amylopectin synthesis [10,12]. The inactivation of BEIIb resulted in the *amylose extender* (*ae*) mutant-containing starch molecules with longer amylopectin chains and fewer branches, increasing the amylose content, changing the crystalline pattern of starch from A-type to B- or C-type and producing an opaque seed phenotype in rice [10,12,36,37,38], whereas no such change has been so far reported in the *be1* and *be2a* mutants [11,39]. In addition, mutations in *BEIIb* gene altered the interactions of starch biosynthetic enzymes and changed the formation of multi-enzyme complexes in maize [24,25], barley [28], and rice [30]. These observations imply that BEIIb plays a specific role in the formation of multi-enzyme complexes in amylopectin synthesis. However, there is little information concerning the dynamic change in starch biosynthetic isozyme complexes at different developmental stages of rice endosperm. In addition, most studies on protein complex formation were focused on *japonica* cultivars [30,31,32], which have almost inactive SSIIa [40]. Therefore, whether and how novel protein complexes are present in *indica* rice with active SSIIa isozyme remains unknown.

In a previous study, we investigated the changes in starch fine structure and functional properties in *indica* rice variety IR36 (wild type, WT) and its BEIIb-deficient mutant with reduced BEIIb level (*be2b*) during three typical developmental stages from 5 to 15 DAF [41]. In the present study, we analyzed dynamic changes in total protein expression profiles and multi-enzyme complexes in the same materials by gel-filtration chromatography, co-immunoprecipitation, and Western blot analysis. In addition, proteomic analysis was further conducted to confirm the putative large complexes (>400 kDa) to reveal the presence of other proteins in the complexes and differentially expressed proteins between WT and *be2b* at different developmental stages. Finally, the essential role of protein phosphorylation and protein kinases in multi-protein complex formation was discussed. These results not only provide valuable insights into the roles of BEIIb in the formation of multi-enzyme complexes, but also provide a novel understanding of the complex network of starch biosynthetic enzymes involved in starch accumulation in developing endosperm.

## 2. Results

### 2.1. Accumulation of Starch Biosynthetic Related Enzymes (SSREs)

**Figure 1:**
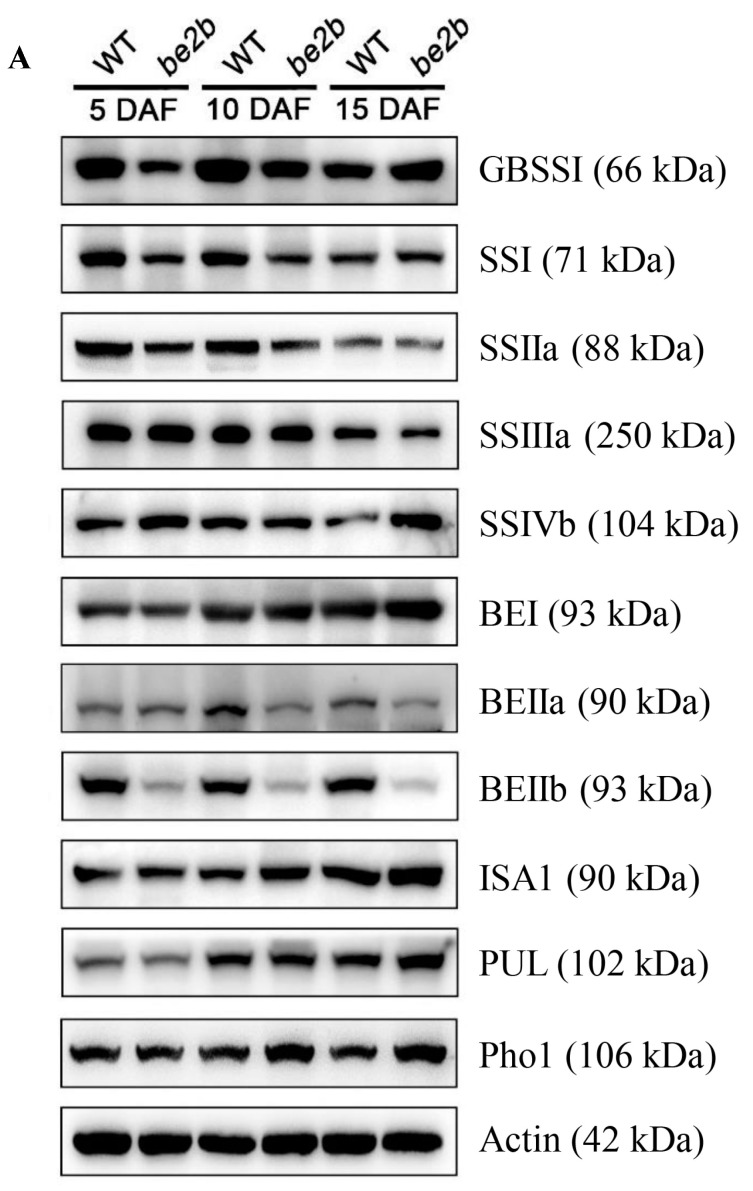

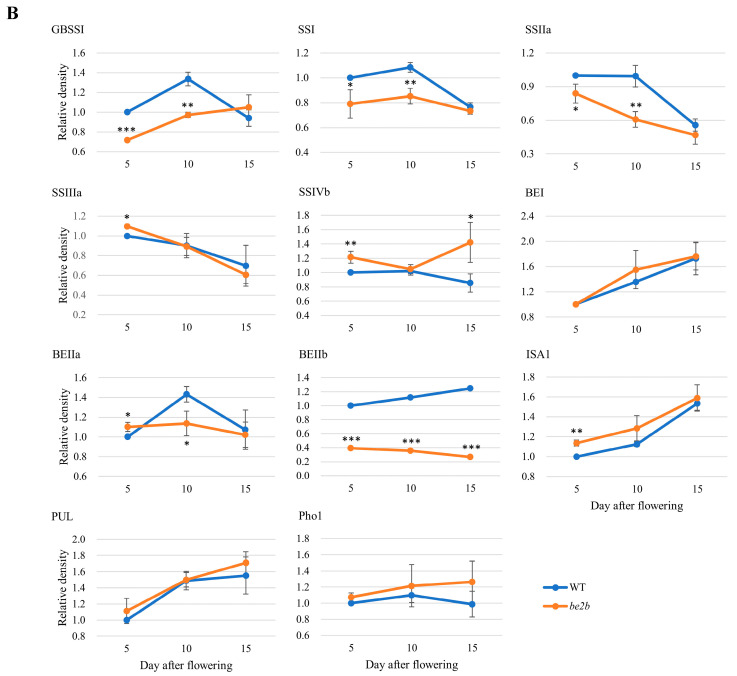
The expression of starch biosynthetic related enzymes (SSREs) during different grain-filling stages (5 DAF, 10 DAF, 15 DAF) in wild type (WT) and *be2b* mutant: (**A**) Total proteins were separated by SDS-PAGE. Each lane was loaded with 50 μg of proteins. Gels were analyzed by Western blotting using the indicated isozyme-specific antibodies. The anti-actin antibody is used as a loading control. Results shown are representative of two biological replicates. (**B**) Relative density of Western blotting results measured by Image J. Data are Mean ± SD from two biological replicates. The asterisks indicate statistical significance between WT and *be2b*, as determined by the Student’s *t*-test (*, *p* < 0.05; **, *p* < 0.01; ***, *p* < 0.001).

To investigate the effects of BEIIb deficiency on other starch biosynthetic-related enzyme (SSRE) accumulation in rice endosperm at different developmental stages, total proteins were isolated from the WT and *be2b* mutant [41], and 11 antibodies from GBSS, SS, BE, DBE, and Pho classes were applied to detect the expression of SSREs (Figure 1A). As shown in Figure 1B, quantification of Western blot bands revealed SSREs were differentially accumulated in WT and *be2b* at different grain filling stages. It is expected that the accumulation of BEIIb significantly decreased in *be2b*, and a significant reduction in SSI and SSIIa was also observed. However, the number of SSIVb, BEI, ISA1, PUL, and Pho1 was up-regulated in *be2b*. The total GBSSI level was lower in *be2b* than in WT at 5 and 10 DAF, but higher at 15 DAF, while SSIIIa and BEIIa were higher at 5 DAF, but subsequently decreased. 

The relative levels of SSREs vary with the dynamic changes in endosperm development in both WT and *be2b*; although, the protein levels fluctuated within a narrow range (less than twofold) as estimated by immunoblot (Figure 1B). In WT, BEI, BEIIb, ISA1, and PUL increased in the steady state level over development, while SSIIa and SSIIIa decreased, and the accumulated levels of GBSSI, SSI, SSIVb, BEIIa, and Pho1 peaked at the mid developmental stage. In the *be2b* mutant, the dynamic changes in some enzymes were strongly affected by a dramatic reduction in the BEIIb level. GBSSI and Pho1 displayed an upward trend from 5 to 15 DAF, and the relative level of SSIVb fell from 5 to 10 DAF, then increased. The changing pattern of other enzymes, SSI, SSIIa, SSIIIa, BEI, BEIIa, ISA1, and PUL, were identical to that of WT.

### 2.2. Molecular Weight Distribution of SSREs

**Figure 2:**
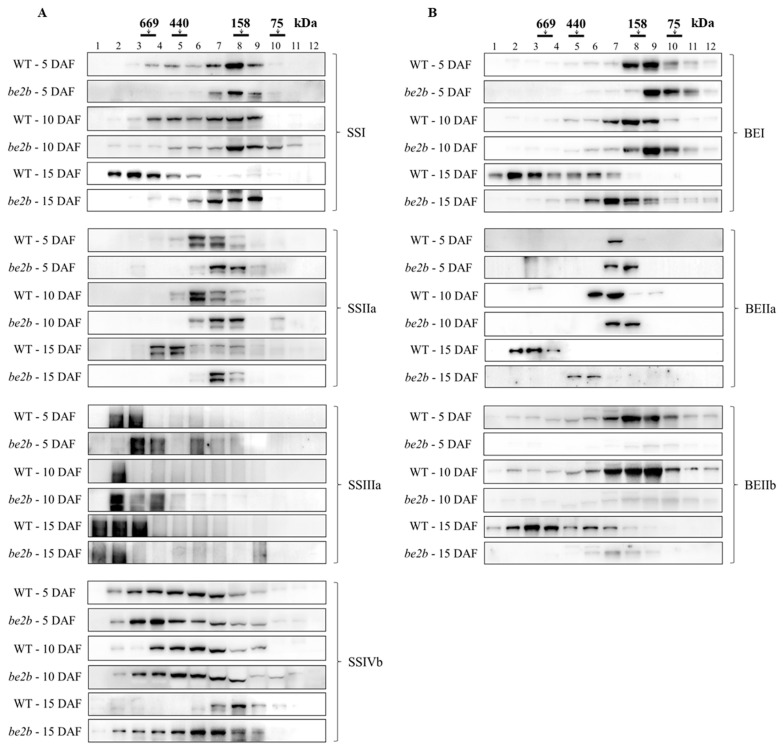

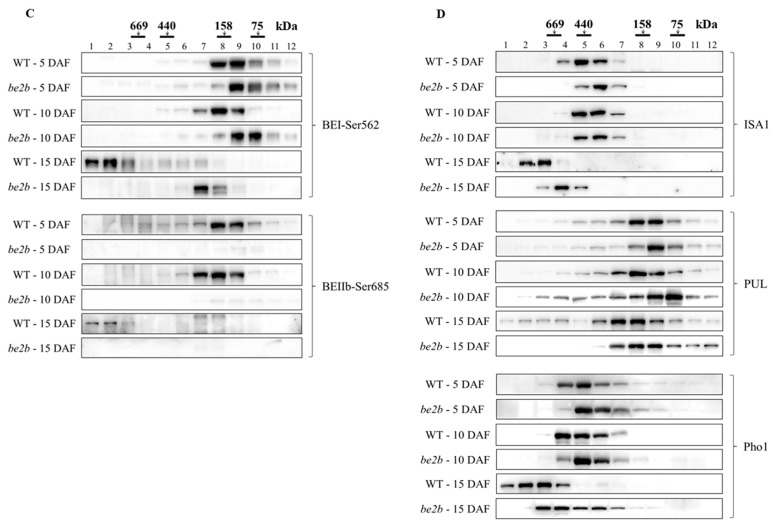
Molecular weight distributions of SSREs during different grain-filling stages (5 DAF, 10 DAF, 15 DAF) in wild type (WT) and *be2b* mutant. Fractions 1–12 obtained after gel filtration chromatography of soluble proteins extracted from the developing seeds were separated by SDS-PAGE prior to Western blotting with indicated isozyme-specific antibodies. Each lane was loaded with 5 μL of 10-fold concentrated fractions. The molecular weight of protein standards is shown at the top (black bars): (**A**) Western blotting of SS isozymes. (**B**) Western blotting of BE isozymes. (**C**) Western blotting of phosphor BE isozymes. (**D**) Western blotting of DBE isozymes and Pho1.

Soluble proteins extracted from developing seeds of WT and *be2b* at different grain-filling stages were separated by gel-filtration chromatography. The eluted fractions were denatured and analyzed by Western blotting using enzyme-specific antibodies to examine possible changes in the aggregation state of the main SSREs involved in amylopectin biosynthesis. In addition, the phosphorylation status of BEs was also examined with site-specific phosphopeptide antibodies (BEI-Ser562, BEIIb-Ser685) identified previously [42]. Figure 2 shows that while some proteins could be detected at smaller than their expected monomeric size derived from partial fragments of isozymes, all starch biosynthetic proteins analyzed were eluted at higher molecular weights, suggesting the existence of protein multimerization in all samples. As expected, faint signals of the BEIIb band (ca. 87 kDa) were observed in the fractions of *be2b* compared with WT. In comparison with BEI and BEIIb (Figure 2B), phosphoproteins showed elution patterns with similar elution positions but narrower molecular weight ranges (Figure 2C). Although some differences in elution patterns of isozymes were noted at 5 and 10 DAF between WT and *be2b*, striking differences were observed at 15 DAF except SSIIIa. The majority of SSI, BEI, and BEIIb were eluted in fractions 2 to 5 (>400 kDa) in WT at 15 DAF, while in the *be2b* mutant, all of these enzymes were eluted mainly in fractions 6 to 9 (100–300 kDa). Similarly, SSIIa, BEIIa, ISA1, PUL, and Pho1 also eluted earlier from the column in WT than those in *be2b* extracts, indicating that these proteins are components of larger complexes in WT rather than in *be2b*. By contrast, SSIVb was eluted in a broad molecular weight range (fractions 2–9, >100 kDa) in *be2b* at 15 DAF, but in a low molecular weight form (fractions 7–9, 100–200 kDa) in WT. 

Considering the dynamic changes in endosperm development, most of the enzymes were eluted in higher molecular weight fractions at later stages of endosperm development (10 and 15 DAF) than at the earlier stage (5 DAF), in both WT and *be2b*. Specifically, most of SSI, BEI, BEIIb, and PUL were eluted in low molecular weight fractions 7 to 9 (100–200 kDa) at 5 and 10 DAF in WT, whereas the amount of these enzymes eluted in fractions 2 to 6 (>300 kDa) was considerably elevated at 15 DAF. SSIIa was mainly detected in fractions 6 and 7 (200–300 kDa) at 5 and 10 DAF in WT, while only a small amount was found in these fractions, and most of them were eluted in fractions 4 and 5 (400–600 kDa) at 15 DAF. BEIIa was detected in fractions 7, fractions 6 and 7, and fractions 2 to 4 (>500 kDa) at 5, 10, and 15 DAF in WT, respectively. ISA1 and Pho1 were found in fractions 4 to 7 (200–600 kDa) at 5 and 10 DAF in WT, whereas they were mainly eluted in fractions 2 and 3 (>600 kDa) at 15 DAF. The elution patterns of SSIIIa in different samples were almost identical; although, the overall trend was that with the development of seed, the high molecular weight fractions were increased. Similar results were also obtained from the *be2b* mutant, indicating an apparent increase in molecular mass or aggregation state of these enzymes with the endosperm development. However, an opposite trend was found in the SSIVb protein; it was inclined to form a larger protein complex at earlier stages in WT, while in *be2b*, the elution patterns of SSIVb were similar among different developmental stages, suggesting SSIVb plays a different role in protein complex formation.

### 2.3. Co-Immunoprecipitation

**Figure 3:**
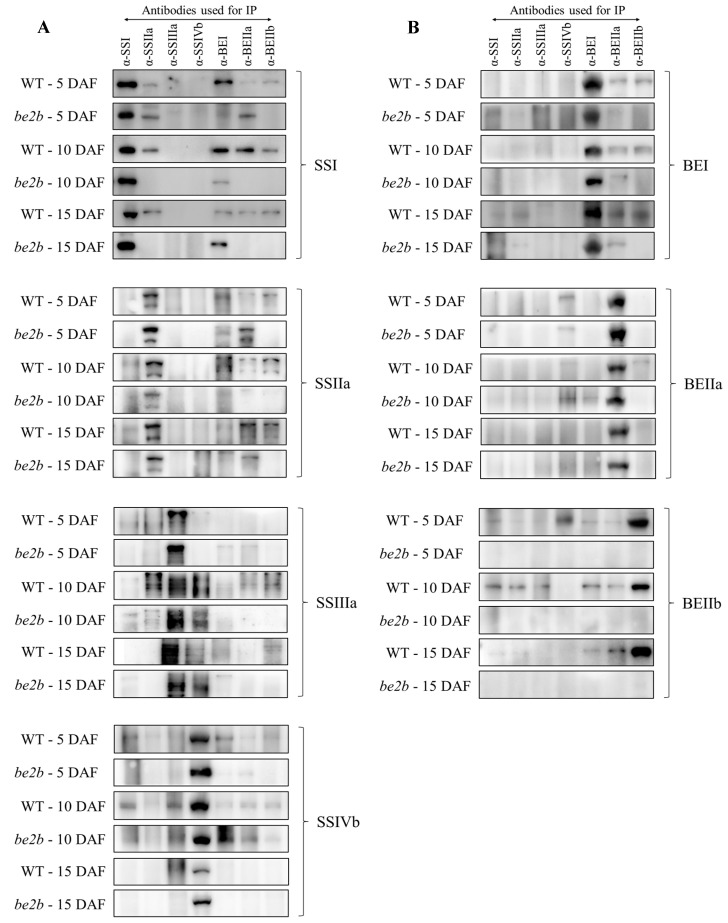

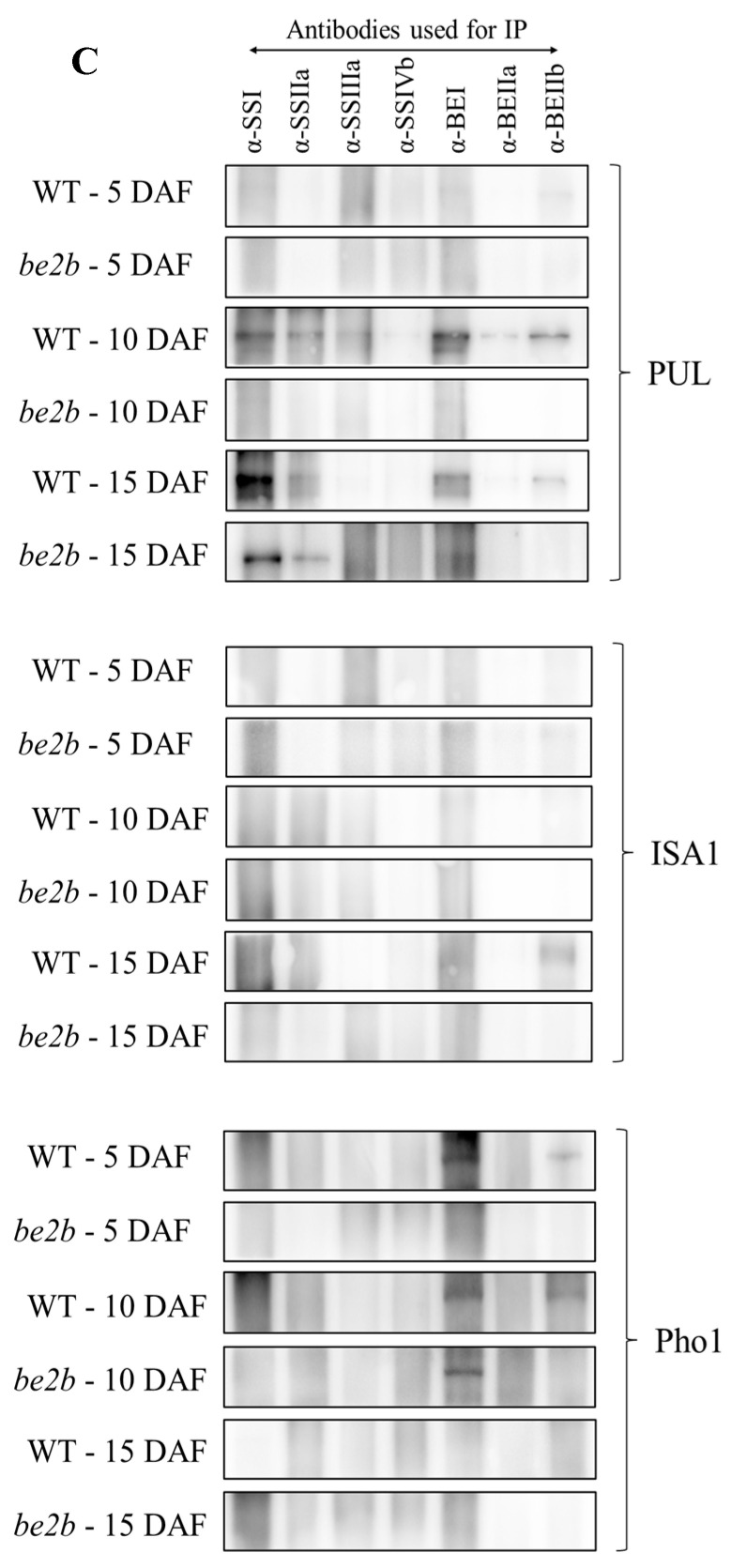
Protein–protein interactions between SSREs during different grain-filling stages (5 DAF, 10 DAF, 15 DAF) in wild type (WT) and *be2b* mutant by co-immunoprecipitation (Co-IP). Soluble proteins extracted from developing seeds were incubated with the indicated isozyme-specific antibodies and protein A magnetic beads. After washing, the captured proteins were released by boiling in a 1× SDS buffer and 5 μL of each supernatant was separated by SDS-PAGE prior to Western blotting. The antibodies used for precipitation as shown in the top panels. The antibodies used for Western blotting are indicated on the right: (**A**) Western blotting of SS isozymes. (**B**) Western blotting of BE isozymes. (**C**) Western blotting of DBE isozymes and Pho1.

In order to investigate the differences in possible interacting partners among SSREs in WT and *be2b* at different developmental stages, soluble proteins were extracted from the developing seeds and co-immunoprecipitation experiments were performed using enzyme-specific antibodies. All of the antibodies used for precipitation (anti-SSI, anti-SSIIa, anti-SSIIIa, anti-SSIVb, anti-BEI, anti-BEIIa, and anti-BEIIb) were able to recognize and precipitate their respective target protein (Figure 3). The results are summarized in Table 1. At the early grain filling stage (5 DAF), no strong pairwise association was detected by reciprocal co-immunoprecipitation in both WT and *be2b*, while weak signals were obtained from reciprocal co-immunoprecipitation experiments for the pairwise interactions BEIIb–SSI, BEIIb–SSIIa, BEIIa–SSIVb, BEIIb–SSIVb, and BEIIb–BEI in WT and only BEIIa–SSIVb in *be2b*. Clear Western blot signals were obtained for only one way in the co-immunoprecipitation experiments in some cases; among such interactions, BEI–SSIIa, SSIIa–SSI, BEIIa–SSI, and BEIIa–SSIIa were commonly observed in WT and *be2b* (first acronym, the antibody used for immunoprecipitation; second acronym, the isozyme subsequently detected by Western blotting).

However, at later stages (10 and 15 DAF), when a higher molecular mass of enzymes was detected in the gel-filtration experiments (Figure 2), strong pairwise associations were obtained by reciprocal co-immunoprecipitation and less weak immunodetections of co-precipitated protein were observed. In WT, BEIIb was clearly co-immunoprecipitated by SSI, SSIIa, SSIIIa, BEI, and BEIIa at 10 DAF and SSI, SSIIa, and BEI at 15 DAF, while no interactions were observed between BEIIb and other enzymes in *be2b* mutant as expected. In addition, interactions among other starch biosynthetic enzymes were reduced in *be2b*, whereas the interaction between BEIIa and BEI was observed by reciprocal co-immunoprecipitation in *be2b* but assessed by a one-side signal in WT at 10 DAF. Pairwise associations detected by reciprocal co-immunoprecipitation were observed for SSIVb–SSIIIa and BEIIa–SSIVb and one-side signals were obtained for SSI–SSIVb, BEI–SSIVb, and BEIIb–SSIVb in WT at 10 DAF. However, at 15 DAF, when SSIVb was eluted in lower molecular weight fractions (Figure 2), only weak pairwise associations for SSIVb–SSIIIa were observed in WT. Interactions of ISA1 with other starch biosynthetic enzymes only occurred between BEIIb and ISA1 at 15 DAF in WT, indicating that ISA1 was not active in multi-enzyme complex formation in rice.

### 2.4. Identification of Large Protein Complex Components

**Figure 4:**
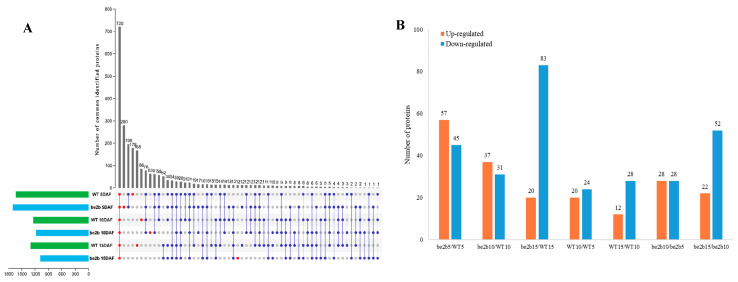
Proteins in high-molecular-weight complexes (>400 kDa) identified by LC-MS/MS during different grain-filling stages (5 DAF, 10 DAF, 15 DAF) in wild type (WT) and *be2b* mutant: (**A**) Upset plot showing the number of proteins that were detected in WT and *be2b* mutant endosperm among the different developmental stages. Red dots indicate proteins identified in all six samples or in only one single sample, blue dots indicate remaining proteins, respectively. (**B**) Number of differentially expressed proteins (DEPs) in relation to *be2b* mutation and developmental stages (3-fold change with *p*-value < 0.05 and PSM > 2).

**Figure 5:**
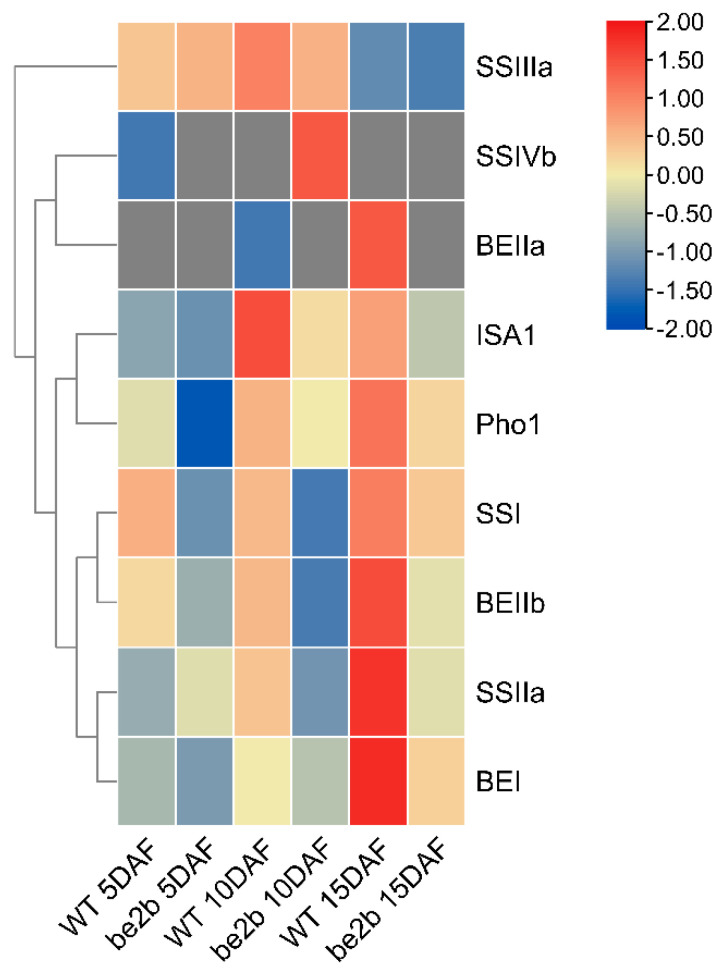
Heat map showing the expression patterns of key enzymes involved in starch synthesis in high-molecular-weight complexes (>400 kDa) identified by LC-MS/MS.

To assess dynamic changes in the composition of high-molecular-weight complexes (>400 kDa) in WT and the *be2b* mutant, fractions 1–5 from two samples at three developmental stages separated by GPC were collected, pretreated, digested, and then analyzed by liquid chromatography–tandem mass spectrometry (LC-MS/MS). In all endosperm samples, we obtained 2524 identities representing 2488 unique proteins (Figure 4A; Table S1). Among these proteins detected, only 720 common proteins were detected in all groups, indicating that the components of large protein complexes involved in starch synthesis differ among developmental stages and between WT and mutant. The number of specific proteins identified in WT and *be2b* peaked at 5 DAF (178 and 280, respectively), followed by 10 DAF (86 and 63, respectively) and 15 DAF (168 and 12, respectively). We further analyzed the expression patterns of key enzymes involved in starch synthesis in high-molecular-weight complexes. It is noted that PUL was not detected by LC-MS/MS due to the lack of MSU ID. As shown in Figure 5, most of the enzymes (SSI, SSIIa, BEI, BEIIa, BEIIb, and Pho1) showed higher expression levels in WT compared to *be2b*, and their abundance was significantly enhanced at 15 DAF. The combination of this proteomic information with the results from GPC and Western blot analysis of soluble protein fractions (Figure 2) suggested that more SSREs assemble into large molecular weight protein complexes in WT, especially at 15 DAF.

To detect possible changes in the composition of high-molecular-weight complexes in relation to developmental stages, differentially expressed proteins (DEPs) were identified by RStudio (version 4.1.2). The DEPs were detected between WT and *be2b* in the corresponding period and the differences between 5 and 10 DAF, 10 and 15 DAF, were also compared between WT and *be2b* (Figure 4B: Tables S2 and S3). Both developmental stages and *be2b* mutation showed a large effect on the formed protein complex. From the perspective of endosperm development, a total of 157 DEPs were differentially expressed between 5 and 10 DAF or 10 and 15 DAF in WT and *be2b*. Among these identified DEPs, some proteins were consistently up-regulated or down-regulated in both WT and *be2b*, while a few enzymes displayed converse regulative patterns between WT and *be2b*. One of the most striking DEPs, isopropylmalate synthase (IPMS1, LOC_Os11g04670), was found to be down-regulated from 5 to 10 DAF and up-regulated from 10 to 15 DAF in WT, but inversely up-regulated from 5 to 10 DAF and down-regulated from 10 to 15 DAF in *be2b*. In comparison with WT, the numbers of up-regulated DEPs in *be2b* were 57, 37, and 20 at 5, 10, and 15 DAF, respectively. In contrast, there were 45, 31, and 83 down-regulated DEPs, respectively. Among these DEPs, two proteins of known function, pyruvate orthophosphate dikinase A (PPDKA, LOC_Os03g31750) and PPDKB (also known as FLO4, LOC_Os05g33570), were of particular interest because both of them were dramatically down-regulated in *be2b* at 15 DAF with a fold-change of 7.3 and 14.7, respectively. 

**Figure 6:**
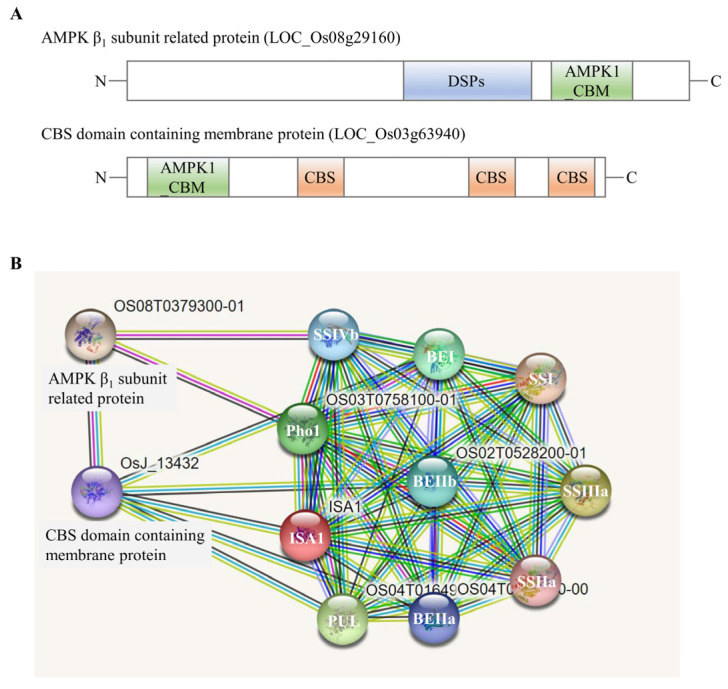
Two protein kinases identified by LC-MS/MS: (**A**) Structure of two protein kinases. (**B**) Potential association networks of two protein kinases and starch synthesis enzymes in rice endosperm from STRING, a database of known and predicted protein interactions. Displayed here is the evidence view, where different line colors represent the types of evidence for the association.

As protein kinases play vital roles in protein complex formation by mediating phosphorylation events, we further delved into these protein kinases identified in high-molecular-weight fractions (>400 kDa) by LC-MS/MS and a total of 29 protein kinases were found (Table S4). Some of them were synchronously present in WT and *be2b*, while other kinases were not. For example, 5′-AMP-activated protein kinase (AMPK) β_1_ subunit-related protein (LOC_Os08g29160) existed in both WT and *be2b* at 5 and 15 DAF, and cystathionine β-synthetase (CBS) domain-containing membrane protein (LOC_Os03g63940) existed in both WT and *be2b* at 5 DAF (Figure 6A). Further prediction of protein interaction with STRING (http://String-db.org) revealed the potential association between AMPK β1 subunit-related protein, CBS domain-containing membrane protein, and some starch biosynthetic enzymes in rice (Figure 6B). In addition, the number of protein kinases found in high-molecular-weight fractions in WT and *be2b* peaked at 5 DAF, followed by 10 DAF and 15 DAF (Table S4).

## 3. Discussion

**Figure 7:**
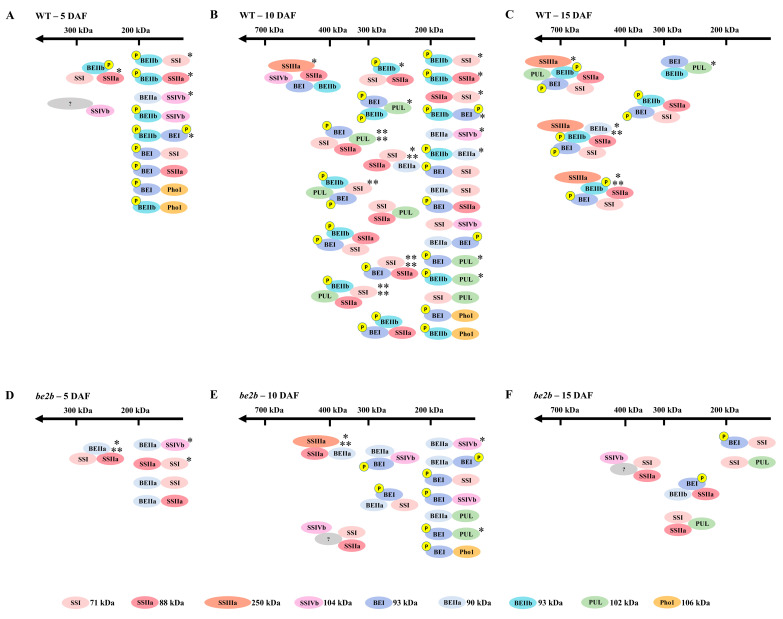
Schematics of speculated starch biosynthetic protein complexes during different grain-filling stages (5 DAF, 10 DAF, 15 DAF) in wild type (WT) and *be2b* mutant: (**A**) WT–5 DAF; (**B**) WT–10 DAF; (**C**) WT–15 DAF; (**D**) *be2b*–5 DAF; (**E**) *be2b*–10 DAF; (**F**) *be2b*–15 DAF. SS isozymes are in red, BE isozymes are in blue, DBE isozymes are in green, Pho1 is in yellow, and phosphate groups are indicated in a small circle. Single, double, triple, and quadruple asterisks indicate the formation of protein complexes confirmed in this study, previously reported by Crofts, et al. [29], Chen and Bao [43], Crofts, et al. [30], and Chen, et al. [44], respectively. Monomeric molecular weights of each isozyme are indicated at the bottom. Note that the schematics do not intend to imply specific details of stoichiometry or direct contacts within the complexes since both direct and indirect physical interactions could be detected by co-immunoprecipitation.

Starch biosynthetic enzymes in cereal are known to interact with each other and to form protein–protein complexes. The main objectives of the current study were to investigate how starch biosynthetic isozyme complexes were altered during grain-filling stages between BEIIb active and deficient rice. Based on the present and past investigations, possible starch biosynthetic protein complexes in WT (Figure 7A–C) and *be2b* (Figure 7D–F) developing endosperm are schematically illustrated. There are some possibilities that starch biosynthetic enzymes interact with each other through glucan-mediated association. However, it seems unlikely since previous studies showed that pre-incubation of wheat and maize amyloplast extracts with glucan-degrading enzymes to remove glucan polymers did not prevent co-immunoprecipitation of SSs, SBEs, and Pho1 [21,25], and had no effect on the GPC analysis [23], suggesting that the formation of multi-enzyme complexes is due to specific protein–protein interactions.

### 3.1. Components of Multi-Protein Complexes Vary at Different Seed Development Stages

The formation and development of starch undergo a complicated physiological and biochemical process in cereal endosperm [45]. It is clear that the structure and composition of starch granules differ during endosperm development in wheat [46,47], barley [48], maize [49], and rice [41], which can be summarized as an increase in amylose content, a decrease in ordered degree, and changes in the amylopectin chain length distribution. All of these changes in starch structure could be explained by the dynamic expression pattern of genes related to starch biosynthesis [1], giving rise to a different accumulation of enzymes, as described above (Figure 1). These enzymes were demonstrated to display as a complex form through post-translational modifications [50]. Consequently, we attempted to reveal how these protein complexes existed in rice endosperm during seed development, which may facilitate the understanding of starch biosynthetic protein complex formation and the ensuing starch structure and properties.

At the early phase of seed development (5 DAF), when endosperm starch begins to accumulate, most of the enzymes were eluted in low molecular weight fractions in both WT and *be2b* as demonstrated by GPC and Western blot analysis (Figure 2). Accordantly, no strong pairwise association was detected by reciprocal co-immunoprecipitation in rice endosperm at 5 DAF (Figure 3; Table 1), suggesting that proteins weakly associate to form small complexes (Figure 7A,D). However, the immunoblotting signal of SSIVb detected in high-molecular-weight fractions was stronger at 5 DAF compared with later stages (Figure 2A). At the mid-phase of seed development (10 DAF), although no remarkable difference was seen in the position of eluted fractions compared with 5 DAF, the number of proteins in higher molecular-weight fractions was visibly elevated, except for SSIVb (Figure 2). This stage involved active protein–protein interaction in rice endosperm as strong pairwise associations were obtained by reciprocal co-immunoprecipitation and less weak immunodetections of co-precipitated protein were observed (Figure 3 and Figure 7B,E; Table 1). At the late phase of seed development (15 DAF), when endosperm starch rapidly increased, there was a notable shift in the elution patterns of the majority of soluble proteins, suggesting an apparent increase in the aggregation state of these enzymes (Figure 2 and Figure 7C,F); although, no increase was observed in pairwise associations obtained by co-immunoprecipitation (Figure 3; Table 1), which was likely due to the steric inhibition effects emanated from the large molecular mass protein complex. These findings were consistent with previous results from developing wheat endosperm [21] and maize endosperm [24] in which the eluted SS and BE activity in high-molecular-weight fractions could only be detected at the later stages. Although the exact mechanism of SSIVb in rice endosperm starch biosynthesis remains unknown, it showed an opposite elution pattern from other patterns during endosperm development, suggesting its possible role in the interaction with other enzymes and in the formation of large complexes to accomplish its activity at the very early stage of endosperm development (Figure 7A). In fact, SSIV is known to play a significant role in the initiation step of starch synthesis involved in MOSs extension and starch granule control in barley [51], wheat [52], and *Arabidopsis* [53,54]. In rice, the mutation of SSIVb did not show a major impact on the starch structure, but the loss of both SSIVb and SSIIIa resulted in opaque seeds with spherical starch granules, suggesting that SSIVb and SSIIIa are key enzymes affecting starch granule morphology [55]. Intriguingly, in the *be2b* mutant, the elution patterns of SSIVb were similar among different developmental stages (Figure 2A). The increased SSIVb in high-molecular-weight complexes might be regulated by the deficiency of BEIIb (see below). Hence, further investigation of enzyme elution patterns and protein–protein interactions in rice mutant lines lacking SSIVb is necessary to gain a more comprehensive understanding of starch synthesis. 

The dynamic changes were further emphasized by proteomic results of high-molecular-weight fractions in which most of the enzymes, including SSI, SSIIa, BEI, BEIIa, BEIIb, and Pho1, showed increased trends from 5 to 15 DAF (Figure 5), indicating these proteins were inclined to form a larger complex at a later developmental stage. IPMS1 was characterized as a DEP in relation to developmental stages, which showed an inverse regulation pattern between WT and *be2b*. IPMS1 was recently identified to regulate seed vigor involved in starch hydrolysis, glycolytic activity, and energy levels [56]. Further work is needed to investigate the effects of BEIIb deficiency on different regulative patterns, which will aid in the understanding of the role of IPMS1 in multi-enzyme complex formation and carbohydrate metabolism.

### 3.2. Effects of BEIIb Deficiency on Multi-Enzyme Complex Formation in the Developing Rice Seed

Protein–protein interactions of starch biosynthetic enzymes are thought to be an important mechanism for efficient starch synthesis [57]. Moreover, studies on mutants that lack starch biosynthetic enzymes have indicated the importance of the formation of starch biosynthetic protein complexes because they could maintain minimal starch biosynthesis by the recruitment of other starch biosynthetic isozymes and the formation of alternative protein complexes; although, the loss of specific starch biosynthetic enzymes may slow starch biosynthesis [33]. Rice mutant seeds with all three major SS activities reduced (*ss1^L^*/*ss2a^L^*/*ss3a*) complemented the composition of protein complexes within the same enzyme family (SS isozyme) to maximize the storage of photosynthetic products such as starch [32]. The substitution of starch biosynthetic protein complexes in BE deficient mutant of maize [24,25], barley [28], and *japonica* rice [30] could also be a similar phenomenon. Based upon previous experimental results, differences in the component of protein complexes between normal rice (WT) and the BEIIb deficiency mutant (*be2b*) in *indica* background with the active SSIIa isozyme were suspected (Figure 7). 

Western blot analysis of SDS-PAGE gels of total proteins from rice endosperm shows that *be2b* used in our study showed a substantial reduction in BEIIb as well as SSI and SSIIa, whilst the protein levels of SSIVb, BEI, ISA1, PUL, and Pho1 were up-regulated compared with WT (Figure 1). Accordingly, the reduction in SSI activity is always accompanied by a deficiency of BEIIb in rice [12,58]. The combined GPC and Western blot analysis of soluble protein involved in amylopectin biosynthesis revealed that the elution pattern of major starch biosynthetic enzymes was altered in *be2b*. In *be2b*, SSI, SSIIa, SSIIIa, BEI, BEIIa, ISA1, PUL, and Pho1 were eluted in lower molecular-weight fractions, and the number of monomeric enzymes (fractions 9–12, <100 kDa) increased compared to WT, especially at 15 DAF (Figure 2). Taking co-immunoprecipitation results into account, associations among these enzymes of *be2b* were weaker than that of WT in the corresponding period (Figure 3; Table 1), indicating that the reduction in BEIIb may either reduce the formation or stability of the protein complex consisting of all these enzymes. Consequently, a considerable decrease in the number of protein complexes at all development stages was hypothesized in *be2b* compared with WT (Figure 7). In addition, no large protein complex containing more than five proteins existed in the *be2b* endosperm, even at 15 DAF (Figure 7F), implying a significant role of BEIIb in protein complex formation. By contrast, SSIVb in *be2b* rice was eluted in earlier fractions than WT and was present in a broader molecular weight range. In addition, the interactions of BEI–SSIVb and BEIIa–SSIVb were stronger in *be2b*, implying alternative protein complexes may form to compensate for BEIIb deficiency. These observations were different from previous studies in *ae^–^* mutants of maize [24] and *japonica* rice [30], which reported the SSI–SSIIa–BEIIb trimeric protein was substituted by the compensatory effects of BEI/BEIIa/Pho1 and BEIIa, respectively, to form the altered complexes. Maize BEI and *japonica* rice BEIIa showed similar changed elution patterns in *be2b* with an increased ratio in the 200–300 kDa fraction (the trimeric protein complex elutes) and broader eluded molecular weight range [24,30]. However, neither BEI nor BEIIa in *be2b* investigated in our study displayed this alteration in molecular weight distribution. Taken together, although BEIIb deficiency leads to a similar altered amylopectin fine structure with less amylopectin short chains and more amylopectin long chains, giving rise to increased gelatinization temperature and amylose content as a result of reduced amylopectin biosynthesis in maize [25], *japonica* rice [58], and *indica* rice [41], the underlying mechanism involved in the synthesis of such *ae* starch may differ as judged by distinct compensatory effects. Our present results suggested that SSIVb likely complemented the role of BEIIb in *be2b* as it was observed in higher molecular-weight protein complexes and had enhanced Western blot signals by co-immunoprecipitation (Figure 2A and Figure 3A), which were analogous to that of BEI/BEIIa/Pho1 in the maize *ae^–^* mutant [24] and BEIIa in the *japonica* rice *ae^–^* mutant [30]. However, neither SSI nor SSIIa associated with SSIVb in *be2b* to form the trimeric protein complex substituted for SSI–SSIIa–BEIIb. The likely explanation is that other potential proteins might bridge the binding of SSIVb to SSI–SSIIa to form a larger alternative protein complex and enhance the association among other starch biosynthetic enzymes to maintain starch biosynthesis in *be2b* (Figure 7E,F). Several enzymes and novel non-enzymatic proteins, including MFP1 [59], PII1 [60], PHS1, and PTST2 [53], were identified to interact with SSIV in *Arabidopsis*. Recently, Zhang, et al. [61] found that carbohydrate-binding module 48 (CBM48) domain-containing protein, FLO6, had a physical association with SSIVb in rice. In addition, correlation analysis between model fitting parameters of amylose and amylopectin chain-length distributions and ratios of the protein content of enzyme pairs revealed that rice SSIVb functionally interacted with SSI, SSIIa, BEI, BEIIb, ISA1, and PUL [62]. Further analyses of the possible interacting partners of SSIVb, the major SSIV isozymes in rice endosperm, will give a better understanding of protein complex formation and starch biosynthesis.

Proteomic evidence of high-molecular-weight fractions from GPC showed that most of the enzyme levels (SSI, SSIIa, BEI, BEIIa, BEIIb, and Pho1) significantly decreased in *be2b* compared with WT (Figure 5), which supported the conclusion that those enzymes are present in a complex with BEIIb. Both PPDKA and PPDKB were significantly down-regulated in the *be2b* mutant at 15 DAF. PPDK catalyzes the formation of the CO_2_ acceptor phosphoenolpyruvate (PEP) from pyruvate, which is most well known as a photosynthetic enzyme in C_4_ plants [63], and the PPDKB deficient mutant caused by T-DNA insertion showed a white-core endosperm, suggesting that the essential function of PPDKB in modulating the carbon flow during grain filling [64]. It was reported that PPDK1 and/or PPDK2 existed in high molecular mass forms that require multiple starch biosynthetic enzymes as both of them were present in the partially purified C670 fraction and identified in the eluate from the SSIIIHD affinity column [23]. In addition, PPDK was identified by nano-LC-MS/MS in rice starch granule-bound proteins whose composition was thought to reflect the composition of the starch biosynthetic protein complex [30]. These findings, taken together with the proteomic investigations of high-molecular-weight fractions from GPC, suggest that PPDKA and PPDKB might also be assembled into large molecular-weight protein complexes in rice for starch biosynthesis. It can be speculated that PPDKA and PPDKB may participate in the complex with the combination of BEIIb because both of them were remarkably down-regulated in *be2b* as described above. Further analyses are required to detect the direct evidence of such protein–protein interactions using antibodies against PPDK, to validate the prediction of the composition of protein complexes. 

### 3.3. The Essential Role of Protein Phosphorylation and Protein Kinases in Multi-Protein Complexes Formation

It is now well accepted that the ability of some key enzymes to form physical interactions with other proteins and their catalytic activity is modulated by protein phosphorylation [50,65]. The phosphorylation status of BEs was also confirmed in our study (Figure 2C), and their narrower eluted molecular weight ranges compared with BEI and BEIIb reflected a regulatory mechanism of protein phosphorylation in rice endosperm.

Protein phosphorylation is a reversible process regulated by a series of protein kinases [57]; however, the kinases present in starch biosynthetic enzyme complexes remain to be defined. One of the protein kinases eluted from high-molecular-weight fractions and characterized by LC-MS/MS in the present study was AMPK β_1_ subunit-related protein, which contains a dual specificity protein phosphatases (DSPs) domain and an AMPK1_carbohydrate-binding module (AMPK1_CBM) domain (Figure 6A). DSPs regulated the activity of their substrates by dephosphorylating threonine/serine and/or tyrosine residues [66]. The AMPK1_CBM domain showed high similarity to CBM20, CBM48, and CBM53 [67], whose surface revealed a carbohydrate-binding pocket to help the kinases, as well as AMPK1_CBM-associated enzymes, bind to starch. AMPK is a ubiquitously expressed, highly conserved heterotrimeric kinase complex with an α (catalytic) subunit and regulatory β and γ subunits in eukaryotic animal cells, which works as a cellular energy sensor in glucose and lipid metabolism [68], while the exact role of AMPK in plants and fungi has not been declared yet. Another kinase was CBS domain-containing membrane protein, which also possessed an AMPK1_CBM domain (Figure 6A). We boldly speculated that this kinase might work as a γ subunit of AMPK in rice because it was composed of three tandem repeats of the CBS domain (four CBS in γ subunit), which bound AMP, ADP, or ATP in a competitive manner according to the changes in energy to modulate the activity of AMPK [69]. Consistent with our hypothesis, the prediction of functional protein association networks showed that the protein interaction of two identified kinases can be formed in the rice endosperm (Figure 6B). Interestingly, both of them also associated with several starch biosynthetic enzymes (Figure 6B), indicating they might be essential components of multi-enzyme complexes in starch biosynthesis. It is difficult to verify such associations by conventional methods because the interaction between kinase and starch biosynthetic enzymes was weak and transient, and the investigation might suffer from the low water solubility of protein. Therefore, the employment of emerging approaches such as TurboID-based proximity labeling technology [70], will shed new light on the key roles of regulatory kinases in multi-protein complexes formation and starch synthesis.

In conclusion, the evidence presented in this article clearly shows that the components of multi-protein complexes changed among different developmental stages, as well as between BEIIb active and deficient rice. With the development of endosperm, most of the enzymes tend to form larger protein complexes except for SSIVb. The converse distribution pattern of SSIVb may be attributed to its vital role in the initiation step of starch synthesis. However, in the *be2b* mutant, BEIIb is not present in any complex, which results in the reduced molecular weight of protein complexes, and a considerable decrease in the number of protein–protein interactions. The large and coordinated protein complexes formed in normal rice endosperm coincide with the fact that starch biosynthesis becomes accelerative during seed development. At the late stage, the diffusion rates of enzymes and substrates are expected to decelerate compared with the relatively aqueous environments during the early stages of seed development, so more efficient machinery is necessary. However, the massive changes in the formation of multi-enzyme complexes in *be2b* endosperm hindered efficient starch synthesis and resulted in the production of modified amylopectin with reduced branching frequency, and longer DP of branches during seed development [41], since other enzymes could only supplement but could not substitute for the crucial role of BEIIb. Again, our results emphasized the importance of the temporal and spatial coordination of multiple starch biosynthetic enzymes for efficient starch synthesis [29,33]. The loss of any components of protein complexes at any stage of seed development will potentially endow starches with altered structure. Hence, the identification of novel proteins involved in multi-enzyme complexes will not only be required to gain further insight into the mechanisms responsible for starch synthesis, but also to provide new targets for improving the quality and yield of rice grains.

## 4. Materials and Methods

### 4.1. Plant Materials and Growth Conditions

*Oryza sativa* subsp. *indica* cv. IR36 (wild type, WT) and a BEIIb-deficient mutant (*be2b*) were used in this study [41]. Both of them were planted at the experimental farm of Zhejiang University, Hangzhou, China, during the summer months under natural conditions. Individual panicles were labeled during flowering and developing seeds were handpicked from fresh plants at 5, 10, and 15 DAF, then immediately frozen in liquid nitrogen and stored at −80 °C. Husks and seed coats were removed before further analysis.

### 4.2. Protein Extraction

Total protein and soluble proteins were extracted on ice as previously described [29]. After extraction, samples were centrifuged at 14,000× *g* at 4 °C for 40 min. The supernatant was collected, and the protein concentration was estimated using a NANODROP 2000 spectrophotometer (Thermo, San Jose, CA, USA) before further analysis.

### 4.3. Gel Permeation Chromatography

Soluble protein was filtered through a 0.22 μm syringe filter to remove large particles and injected into a 500 μL sample loop, prior to fractionation by gel permeation chromatography (GPC) using Superdex 200 resin packed in a 10/300 column connected to an ÄKTA^TM^ *prime plus* chromatography system (GE Healthcare, Chicago, IL, USA). The column was routinely calibrated using commercial gel filtration calibration kits from 75 to 669 kDa (GE Healthcare, USA) and equilibrated with 10 mM HEPES-KOH, pH 7.5, 100 mM NaCl, at a flow rate of 0.4 mL min^−1^. Fractions of 0.8 mL were collected when elution volume was 6.6 mL and concentrated 10-fold using an Amicon Ultra 30K centrifugal filter unit (Merck Millipore, Darmstadt, Germany). Concentrated samples were further supplemented with SDS–PAGE sample loading buffer (Beyotime Biotechnology, Haimen, China) following the manufacturer’s instructions before SDS-PAGE and Western blotting.

### 4.4. Co-Immunoprecipitation

Co-immunoprecipitation experiments were conducted as described in Crofts, et al. [29] with some modifications. Soluble proteins, extracted as described above, were incubated with isozyme-specific antibodies and protein A magnetic beads (New England Biolabs, Ipswich, MA, USA). After extensive washing with phosphate-buffered saline (PBS) (137 mM NaCl, 10 mM Na_2_HPO_4_, 2.7 mM KCl, and 1.8 mM KH_2_PO_4_ at pH 7.4), bound proteins were released by boiling in 1× sodium dodecyl sulfate (SDS) buffer (Beyotime Biotechnology, China) and 5 μL of each supernatant was analyzed by Western blotting.

### 4.5. Western Blotting

Proteins were resolved by 8% SDS-PAGE (SDS-polyacrylamide gel electrophoresis), and transferred onto polyvinylidene fluoride (PVDF) membranes using a transblotter. Western blotting procedure was carried out according to the method of Crofts, et al. [71]. Anti-rice GBSSI, SSI [72], SSIIa, SSIIIa [71], SSIVb [29], BEI, BEIIb [73], PUL [74], and Pho1 [16] antibodies were kindly gifted by Prof. Naoko Fujita (Akita Prefectural University, Akita, Japan). Anti-rice BEIIa (the polypeptide CAGAPGKVLVPG) and ISA1 (the polypeptide CEPLVDTGKPAPYD) [29] antibodies were produced by company (HuaAn Biotechnology Co., Ltd., Hangzhou, China). Site-specific phosphopeptide antibodies (BEI-Ser562, BEIIb-Ser685) were produced as previously described [50]. Anti-beta-actin antibody was purchased from Sigma-Aldrich (St. Louis, MO, USA). Western blot results were quantitated using the Image J software [75].

### 4.6. Protein Preparation, Digestion, LC-MS/MS, and Data Analysis

High-molecular-weight fractions (fractions 1–5, >400 kDa) of 300 µL separated by GPC were mixed together and concentrated 30-fold using an Amicon Ultra 30K centrifugal filter unit (Merck Millipore, Germany). FASP digestion and LC-MS/MS analysis were performed according to the method described by Pang, et al. [50]. Raw data were analyzed with Proteome Discoverer (version 2.4) and were compared with the rice database. Searches were performed using a fragment tolerance of 0.10 Da, and a parent tolerance of 20 ppm, with carbamidomethyl of cysteine as a fixed and oxidation of methionine as variable modifications. Trypsin/P was specified as the enzyme, with maximum missed cleavages allowed of up to 2. Protein identifications were accepted if they achieved a minimum of 1 peptide per protein and a false discovery rate (FDR) of <1%.

### 4.7. Statistical and Bioinformatic Analyses

For differentially expressed proteins (DEPs) analysis, quantified protein abundances were integrated and then normalized by the DEP (Differential Enrichment analysis of Proteomics data) package (version 1.8.0) in RStudio (version 4.1.2) as the input for statistical analysis. Candidates that met the following criteria were selected: fold-change > 3, *p* < 0.05, and peptide-spectrum match (PSM) >2. The least significant difference (LSD) multiple range test was conducted for comparison of the mean of samples at *p* < 0.05.

## Figures and Tables

**Table 1:** Comparison of protein-protein interactions among starch synthetic related enzymes during different grain-filling stages (5 DAF, 10 DAF, 15 DAF) in wild type (WT) and *be2b* mutant endosperms determined by co-immunoprecipitation.

Sample		Reciprocal		One Sided ^a^	
		Strong Signal ^b^	Weak Signal ^b^	Strong Signal ^b^	Weak Signal ^b^
5 DAF	WT		BEIIb–SSI BEIIb–SSIIa **BEIIa–SSIVb** BEIIb–SSIVb BEIIb–BEI	BEI–SSI **BEI–SSIIa** BEI–Pho1	**SSIIa–SSI** **BEIIa–SSI** **BEIIa–SSIIa** SSIVb–SSIIIa SSI–SSIVb BEI–SSIVb BEIIa–BEI BEIIa–BEIIb BEIIb–Pho1
	*be2b*		**BEIIa–SSIVb**	**SSIIa–SSI** **BEIIa–SSI** **BEIIa–SSIIa**	**BEI–SSIIa** BEI–SSIIIa BEIIa–SSIIIa
10 DAF	WT	BEIIb–SSI BEIIb–SSIIa **SSIVb–SSIIIa** BEIIb–BEI	SSIIa–SSI BEIIb–SSIIIa **BEIIa–SSIVb** BEIIb–BEIIa	**BEI–SSI** BEIIa–SSI BEI–SSIIa **SSIIa–SSIIIa** SSI–SSIVb BEIIa–BEI SSI–PUL BEI–PUL BEIIb–PUL **BEI–Pho1**	**BEIIa–SSIIa** **SSI–SSIIIa** BEIIa–SSIIIa **BEI–SSIVb** BEIIb–SSIVb SSIIa–PUL SSIIIa –PUL BEIIa–PUL BEIIb–Pho1
	*be2b*	**SSIVb–SSIIIa**	**BEIIa–SSIVb** BEIIa–BEI	**BEI–SSI** **BEI–SSIVb** **BEI–Pho1**	**BEIIa–SSIIa** **SSI–SSIIIa** **SSIIa–SSIIIa**
15 DAF	WT	SSIIa–SSI **BEI–SSI** BEIIb–SSI BEIIb–SSIIa	BEI–SSIIa SSIVb–SSIIIa BEIIb–BEI	BEIIa–SSI **BEIIa–SSIIa** BEIIb–SSIIIa BEIIb–BEIIa **SSI–PUL** BEI–PUL	**BEI–SSIIIa** **BEIIa–BEI** SSIIa–PUL BEIIb–PUL BEIIb–ISA1
	*be2b*	**BEI–SSI**		SSIVb–SSIIIa **SSI–PUL**	**BEIIa–SSIIa** SSI–SSIIIa **BEI–SSIIIa** SSIIa–BEI **BEIIa–BEI** SSIIa–PUL

^a^ The antibody used for immunoprecipitation is shown on the left and the coprecipitated enzyme detected by Western blotting is shown on the right. Same interactions between the wild type and *be2b* mutant are indicated in bold. ^b^ Protein bands in black or dark grey were defined as strong signals, while those in light gray (but clear) were defined as weak signals.

## Data Availability

The data supporting the findings of this study are available within the article and its supplementary materials.

## Supplementary Materials

The following supporting information can be downloaded at: https://www.mdpi.com/article/10.3390/ijms231810714/s1.
