# Correction: Ying et al. Dynamic Change in Starch Biosynthetic Enzymes Complexes during Grain-Filling Stages in BEIIb Active and Deficient Rice. *Int. J. Mol. Sci.* 2022, *23*, 10714

**DOI:** 10.3390/ijms24108583

**Published:** 2023-05-11

**Authors:** Yining Ying, Feifei Xu, Zhongwei Zhang, Piengtawan Tappiban, Jinsong Bao

**Affiliations:** 1 Institute of Nuclear Agriculture Science, College of Agriculture and Biotechnology, Zhejiang University, Zijingang Campus, Hangzhou 310058, China; 2 Hainan Institute of Zhejiang University, Hainan Yazhou Bay Seed Lab, Yazhou Bay Science and Technology City, Yazhou District, Sanya 572025, China

In the original publication [1], there were mistakes in the legend for Figures 2D and 3C. We carelessly copied the legend from Figure 2B to Figure 2D, and “Pho1” was missing in both legends, i.e., ‘Figure 2D Western blotting of BE isozymes’; and ‘Figure 3C Western blotting of DBE isozymes’. However, the real western blots in Figures 2D and 3C are for DBE and Pho1. The correct legends appear below. The authors state that the scientific conclusions are unaffected. This correction was approved by the Academic Editor. The original publication has also been updated.

**Figure 2.** Molecular weight distributions of SSREs during different grain-filling stages (5 DAF, 10 DAF, 15 DAF) in wild type (WT) and *be2b* mutant. Fractions 1–12 obtained after gel filtration chromatography of soluble proteins extracted from the developing seeds were separated by SDS-PAGE prior to Western blotting with indicated isozyme-specific antibodies. Each lane was loaded with 5 μL of 10-fold concentrated fractions. The molecular weight of protein standards is shown at the top (black bars): (**A**) Western blotting of SS isozymes. (**B**) Western blotting of BE isozymes. (**C**) Western blotting of phosphor BE isozymes. (**D**) Western blotting of DBE isozymes and Pho1.

**Figure 3.** Protein–protein interactions between SSREs during different grain-filling stages (5 DAF, 10 DAF, 15 DAF) in wild type (WT) and *be2b* mutant by co-immunoprecipitation (Co-IP). Soluble proteins extracted from developing seeds were incubated with the indicated isozyme-specific antibodies and protein A magnetic beads. After washing, the captured proteins were released by boiling in a 1× SDS buffer and 5 μL of each supernatant was separated by SDS-PAGE prior to Western blotting. The antibodies used for precipitation as shown in the top panels. The antibodies used for Western blotting are indicated on the right: (**A**) Western blotting of SS isozymes. (**B**) Western blotting of BE isozymes. (**C**) Western blotting of DBE isozymes and Pho1.
